# Plastic Contamination in Seabass and Seabream from Off-Shore Aquaculture Facilities from the Mediterranean Sea

**DOI:** 10.3390/jox13040040

**Published:** 2023-10-25

**Authors:** Giacomo Mosconi, Sara Panseri, Stefano Magni, Renato Malandra, Alfonsina D’Amato, Marina Carini, Luca Chiesa, Camilla Della Torre

**Affiliations:** 1 Department of Veterinary Medicine and Animal Science, University of Milan, 26900 Lodi, Italy; giacomo.mosconi@unimi.it (G.M.); sara.panseri@unimi.it (S.P.); luca.chiesa@unimi.it (L.C.); 2 Department of Biosciences, University of Milan, 20133 Milan, Italy; 3 ATS Milano-Città Metropolitana, Veterinary Unit, 20122 Milan, Italy; malandra55@libero.it; 4 Department of Pharmaceutical Sciences, University of Milan, 20133 Milan, Italy; alfonsina.damato@unimi.it (A.D.); marina.carini@unimi.it (M.C.)

**Keywords:** microfibers, farmed fish, marine pollution, seafood, ecotoxicology, ecology

## Abstract

We characterized the presence of plastics in different organs of the gilthead seabream (*Sparus aurata*) and European seabass (*Dicentrarchus labrax*) from some off-shore aquaculture facilities of the Mediterranean Sea. Plastics were detected in 38% of analyzed fish. Higher contamination was observed in fish from Turkey and Greece with respect to Italy, without significant differences between the geographical areas. Plastics accumulated mostly in the gastrointestinal tract and, to a lower extent, in the muscle, which represents the edible part of fish. Based on the particle detected, a maximum amount of 0.01 plastic/g wet weight (w.w.) can occur in muscles, suggesting a low input for humans through consumption. A large portion of the particles identified was represented by man-made cellulose-based fibers. The characterization of the polymeric composition suggests that plastics taken up by fish can have land-based and pelagic origins, but plastics can be introduced also from different aquaculture practices.

## 1. Introduction

Plastic contamination is a widespread environmental problem, since extensive production and especially incorrect disposal are leading to a dramatic release of plastics into natural ecosystems [1]. Plastics have, over time, become an increasing problem for marine ecosystems, accounting for around 60–80% of the total debris in such environments and completely outnumbering natural debris [2].

To date, the adverse impacts of plastic and microplastic (MP) contamination have been extensively investigated in marine organisms [3], while the effects on humans are still to be defined [4]. In particular, a controversial aspect of plastic contamination concerns the potential risks for humans from consuming contaminated fish species [5,6]. Indeed, ingestion through seafood consumption is considered one of the major routes of human exposure to MPs [7] based on the evidence of their widespread and huge presence in different seafood such as fish, mussels, and crustaceans [8,9,10]. Nevertheless, data concerning the accumulation of plastics in fish often refer to their presence in the gastrointestinal tract, while the infiltration/accumulation in edible tissues—such as muscle—in commercial species collected under natural conditions is not sufficiently documented. As well, the ability of plastics to biomagnify through the food chain is still controversial [9]. Moreover, there is mounting evidence that the number of MPs ingested via seafood consumption is well below the intake through inhalation via dust and airborne [11,12,13,14]. Therefore, it is crucial to gather more evidence that can contribute to linking the consumption of seafood containing plastics to actual risk for human health.

Fish, by virtue of its nutritional properties, is fundamental to the human diet. Several studies have shown a relationship between fish consumption and reduced risk of heart disease, cardiac arrhythmias, and diabetes due to their high percentage of n-3 polyunsaturated fatty acids (n3PUFAs) [15]. Among marine fish, gilthead seabream (*Sparus aurata*) and European seabass (*Dicentrarchus labrax*) are two of the most consumed in the Mediterranean area, and more than 20% of total EU aquaculture production is represented by these two species [16]. The first three markets for seabass, Italy, Spain, and France, account for more than 70% of total EU consumption, and the annual *per capita* consumption averages 190 g but exceeds 500 g in some Mediterranean countries (Italy, Spain, Portugal, and Cyprus) [17]. As far as seabream is concerned, Italy is the world’s leading consumer, with 38,626 tonnes of consumption, while Egypt, Turkey, and Tunisia are in second, third, and fourth place, respectively [18,19]. In Italy, seabream and seabass are the first and fourth most consumed fresh fish, respectively, representing 14.8% of the total consumption volume, from young children to the elderly [20]. Seabass has demersal behavior and inhabits coastal waters down to about 100 m depth, but is more common in shallow waters, on various kinds of bottoms, often entering estuaries and sometimes ascending rivers. It is a voracious predator, feeding on small shoaling fish and a wide range of invertebrates including shrimps, prawns, crabs, squids, and mollusks [21]. Seabream is a demersal fish found in a variety of bottoms, including seagrass beds and sandy bottoms, as well as the surf zone. Specimens are common up to depths of about 30 m, but adults may occur at 150 m depth. This sedentary fish occurs either solitary or in small aggregations and is mainly carnivorous and accessorily herbivorous, feeding mostly on shellfish, including mussels and oysters [22].

In the literature, there are few recent studies on the detection of plastics in the two species (Table 1), but they focus only on limited geographical areas. Moreover, most refer to the measurement of plastic content in the gastrointestinal tract (GIT), while the accumulation in different organs, especially in muscle, which is the edible part, is currently overlooked.

Therefore, this work aimed to quantify and characterize the presence of plastics using the Fourier transform infrared microscope system (µFT-IR) in individuals of seabass *D. labrax* and seabream *S. aurata* retrieved from off-shore aquaculture facilities located in different sites of the Mediterranean Sea, namely Italy, Greece, and Turkey. Different organs were analyzed: GIT, liver, and muscle, to clarify the potential translocation from the gut to other districts, particularly the muscle, to assess the potential hazard to human health related to fish consumption.

## 2. Materials and Methods

### 2.1. Sample Collection

A total of 17 individuals of seabasses and 17 seabreams from Italy (N = 6), Greece (N = 18), and Turkey (N = 10) were collected upon arrival to the Milan fish market. Fish coming from Turkey were retrieved from Mediterranean aquaculture plants. This sample is representative of the geographical origin of the fish arriving at the market. Two different collections were performed: one in 2019 (N = 14 fish) and one in 2020 (N = 20 fish). Since the GIT represents the first compartment of plastic accumulation, we analyzed plastics in this tissue from all 34 individuals. A sub-sample was processed for plastic determination also in muscle (N = 13) and liver (N = 7). Details about samples are reported in Table S1.

### 2.2. Sample Preparation

The sample processing included a gutting and filleting step for each fish. For each specimen, the muscle, GIT, and liver were separated. The muscles were weighed, and the average weight was 96 ± 21 g. Various compartments were homogenized with hypersaline solution (1.2 g/cm^3^, filtered through glass fiber filters with a porosity of 1.2 μm of the Whatman GF/C 47 mm type) using a blender and subsequently digested overnight. After the digestion phase, a filtration phase took place using a membrane vacuum pump and the use of nitrocellulose filters with 8 µm porosity (Sartorius 5 mm). Prior to analysis for the quantification and characterization of plastics, the filters were partially digested with 15% hydrogen peroxide (H_2_O_2_) to remove organic materials [32,33].

### 2.3. Quantification and Characterization of Particles Using µFT-IR

The cellulose nitrate filters obtained at the end of the processing were then observed using a stereomicroscope in such a way as to carry out visual sorting and thus discern potential plastic particles and fibers from substances of a different nature. The potential plastic debris were then transferred to new cellulose nitrate filters, also with a porosity of 8 µm. The filters were analyzed using a µFT-IR model Spotlight200i equipped with a Spectrum Two optical microscope (PerkinElmer, Italy) through which it was possible to discern the color, shape, size, and chemical composition of the particles. In the analysis, infrared (IR) radiation was used to obtain the IR spectra of each particle under examination, acquired in the attenuated total reflectance (ATR) mode (32 scans of spectra with wavelengths between 600 and 4000 cm^−^^1^ and a resolution of 4 cm^−^^1^) and then analyzed using Spectrum 10 software, comparing them with standard spectra in the library (PerkinElmer). The comparison took into account a matching score ≥ 0.70 as a reference score to define a reliable degree of similarity between the spectra of the analyzed samples and the spectra present in the database [34,35]. Each particle was also classified according to its shape (fragment–fiber–pellet–film), color, and size. In order to analyze the size of the debris under investigation, scans obtained from previous analyses using Spectrum 10 software were used, which were appropriately measured using ImageJ software and characterized as microplastics (from 1 μm to –<1000 μm), mesoplastics (from 1 mm to −<10 mm), and macroplastics (≥1 cm) following the classification of Hartmann et al. [36]. To prevent any bias due to external contamination by atmospheric particles, all analyses were carried out under a flow cabinet, and, in addition, the operators always wore gloves and a cotton smock during the process. Several filters were also processed in parallel to samples, and any debris detected in this filter was subtracted from the final count (Table S2).

### 2.4. Statistical Analysis

To evaluate significant differences in the extent of particle accumulation among the different tissues, and among fish collected in the different geographic regions, data were statistically analyzed using the GraphPad Prism 8.0.2 software package. Since the accumulation did not follow a Gaussian distribution and the sample size of fish and tissues analyzed was not homogeneous, we applied the Kruskal–Wallis *non*-parametric test, considering *p* ≤ 0.05 as the significant cut-off.

## 3. Results

A total of 111 particles were found in fish tissues and analyzed. Among them, 18 were made of plastics, while the remaining were of *non*-synthetic origin, mostly cellulose and natural polyamide (Table 2 and Table S3).

The results showed that 13 fish were contaminated by plastics, representing about 38% of the total. Comparing the two species, 41% of the seabream and 35% of the seabass were contaminated. An average of 0.51 ± 0.78 plastics/individual was measured. A mean of 1.39 ± 0.65 plastics/individual was measured among fish that ingested plastics.

**Figure 1:**
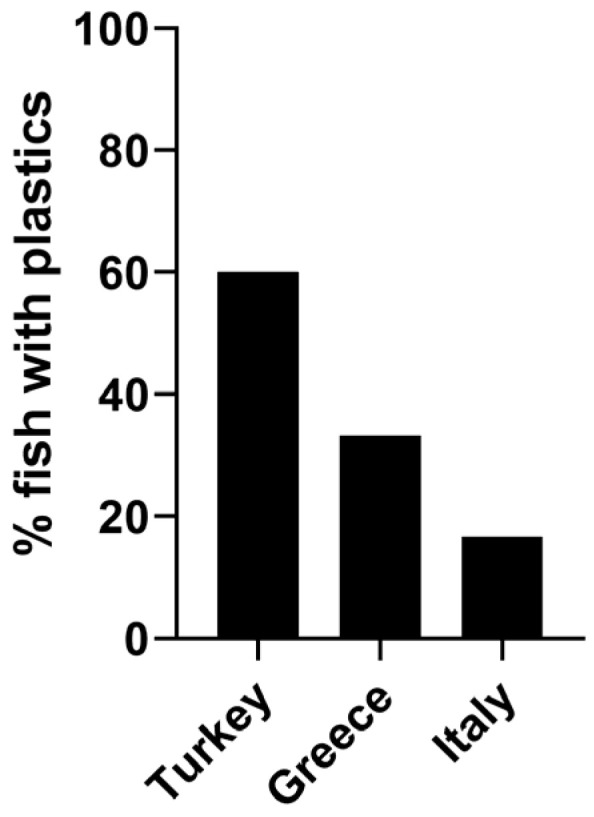
Occurrence of plastic particles in fish related to the geographical provenance.

Different levels of contamination can be observed depending on the geographical region of the origin of the plants, albeit not statistically significant (*p* = 0.2953) (Figure 1). In fish from Turkey and Greece, plastics were detected in 60% and 33% of the total, respectively. Up to 3 items/individual were identified in fish from Greece, while 0–2 items were detected in individuals from Turkey. A much lower incidence was observed in fish from Italy since only one fish was contaminated with one plastic item (17% of the total).

**Figure 2:**
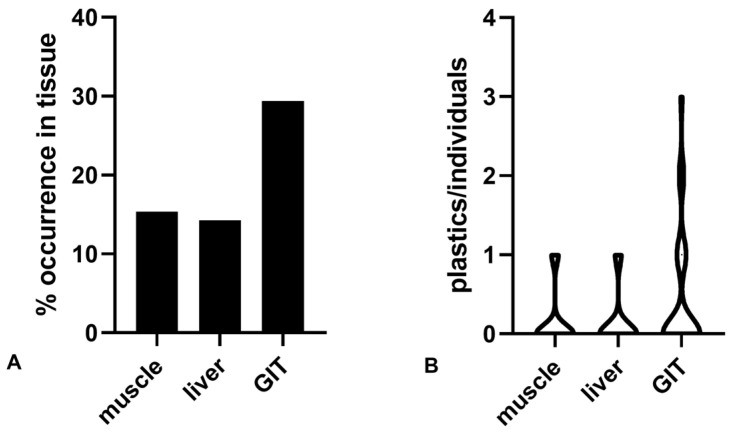
Percentage of occurrence of plastics in tissues analyzed (**A**) and the number of items detected in the same tissue from the same fish (**B**).

Concerning the accumulation in the different tissues, 29.4% of the GIT analyzed contained plastics, with up to 3 particles/individual (Figure 2). Only two muscles contained one item of plastic each, and only one plastic particle was detected in one liver. The accumulation in the three tissues was not significantly different (*p* = 0.4184).

**Figure 3:**
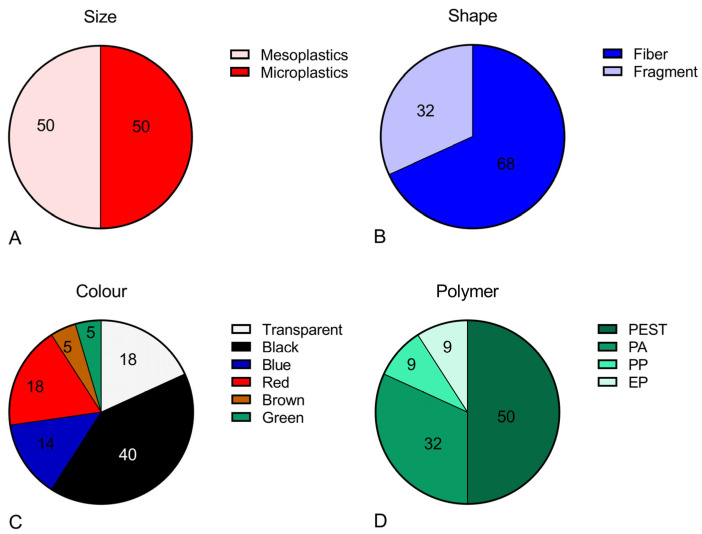
Plastic characterization in terms of size (**A**), shape (**B**), color (**C**), and polymer composition (**D**) in all analyzed fish from Mediterranean Sea.

The qualitative characterization of plastic particles detected showed the same occurrence of MPs and mesoplastics, while macroplastics were not detected (Figure 3A). The most abundant shape was represented by fibers (68%), and the remaining 32% were fragments (Figure 3B). As for the color, the highest percentage (41%) was made of black particles, followed by transparent and red (18%) and blue (14%). A lower percentage of green (5%) and brown (4%) plastics were found (Figure 3C). The polymeric composition of the plastics was in line with the shape since 50% were made of polyester (PEST), followed by 32% of polyamide (PA), while a lower percentage (9%) of polypropylene (PP) and epoxy resin (ER) was found (Figure 3D). Comparing the typology of plastics identified in fish from each country separately, we did not observe relevant differences. A fiber of PEST was identified in the single fish contaminated from Italy, in the mesometric size. In fish from both Turkey and Greece, the most abundant plastic shape was fiber, representing 63% and 89% of the total, respectively. In fish from Turkey, the highest percentage of plastics were microplastics (63%), while in fish from Greece, mesoplastics were more often identified (67%). As far as the polymeric composition is concerned, PEST and PA were the more common polymers. Debris of EP were identified in fish from both countries, while plastic items of PP were detected only in fish from Turkey.

## 4. Discussion

Plastic contamination emerged as a critical issue for aquaculture [37,38,39]. The adverse impacts of MPs, in particular, have been identified in terms of direct toxicity to organisms [10,40,41,42,43], adsorption of environmental pollutants [44], and the spread of pathogens [45]. In this view, the monitoring of plastic contamination in fish from aquaculture systems is important to understand potential point sources and to take actions to mitigate the problem. Our results confirmed that both seabass and seabream from off-shore aquaculture plants are subjected to contamination by plastics, even though the amount of particles detected was low. Comparing our results with the scientific literature, the number of items/individual detected is well below the study conducted by Sánchez-Almeida et al. [23] and Kılıç [27] and lower also to the value obtained by Reinold et al. [25] and Savoca et al. [29]. Our study showed a similar percentage of incidence but a lower number of items/individual with respect to that of Bessa et al. [26]. Conversely, Akoueson et al. [28] found a lower incidence of plastic contamination with respect to ours. This comparison shows that there is a large variability in the extent of plastic contamination in fish from aquaculture systems, not only likely due to the local conditions and to the local level of plastic pollution but also potentially related to the plastic release from each aquaculture facility.

Only two studies reported the occurrence of plastics in the two species collected from the wild. Barboza et al. [30] showed a higher percentage of incidence as well as a higher number of items/individual in seabass collected from the coast of Portugal compared with our results. Also, the study from El-Sayed et al. [31] reported a higher contamination in seabream collected from Alexandria City in Egypt. With the aim to broaden the comparison of our data with those of organisms collected in the wild, we considered the plastic contamination reported in species with similar feeding habits collected in the Mediterranean Sea (Table 3).

Comparing our results with the ones obtained from studies on different fish species collected in the wild, we can see that, in our study, the percentage of plastic occurrence is well below to the one observed in other studies [31,46,47,48,52,53,54]. Conversely, Giani et al. [8] and López-Martínez et al. [50] found a lower occurrence of plastics with respect to us but recorded a higher average number of particles/individuals. The only two studies that reported a lower incidence of plastic were the ones by Capillo et al. [49] and Savoca et al. [51]. Overall, this further comparison seems to suggest that fish from aquaculture are less susceptible of plastic ingestion with respect to wild animals, maybe due to the fact that they are fed and so less prone to take up plastics through active predation, which represent one of the main routes of plastic accumulation in predator species [55].

Concerning the distribution in internal organs, the largest amount of plastics was in the GIT, while a reduced number of debris were detected in liver and muscle, suggesting low translocation from the GIT to these tissues. Among individuals in which we analyzed multiple tissues, plastics were never detected in more than one organ. In general, the highest amount of plastics is found in the GIT, since predatory fish can assume plastics accidentally from the water column or actively through feeding, mistaking with prey, or ingesting contaminated preys [56]. The mechanisms of translocation of plastics from the GIT and their internalization in other organs remain largely unknown [57]. Particles up to 130 μm might be internalized through phagocytosis and endocytosis [58], and passive penetration can occur for nanoplastics [59]. Nevertheless, the presence of larger particles in muscle has been documented, even though the mechanism of internalization is still unknown [30,56]. Therefore, the plastics detected in the muscle tissues, having a size range of 530–1010 μm, could have reached this tissue through adherence to the skin or through skin lesions. This contamination can occur not only at sea but also during fish processing stages such as handling, transport, and packaging, just to mention a few. Moreover, the translocation to internal organs from lesions in the GIT cannot be excluded [30].

Based on the debris detected, we can calculate a mean concentration of 0.0015 particle/g w.w. and a maximum amount of 0.010 particle/g w.w. in muscles. This points out a low intake of particles by humans through fish consumption, since considering about 20.5 kg *per capita* consumption of fish per year [60], a mean uptake of 30.75 particles/y/*per capita* and in the worst-case scenario 250 particles/y/*per capita* were quantified. This range is three times lower to what has been reported for edible fish species including *D. labrax* collected in the Portuguese coast of the Northern Atlantic Ocean (0.054 ± 0.099 particle/g) [30]. On one hand, this result seems to indicate a limited input for humans through the diet; on the other hand, plastics can be transferred to other animals through the production of fish meals, for which all tissues and not just the muscle are used and which are intended not only for feeding cultured fish but also in other types of farming.

Albeit not significantly different, we observed higher contamination in fish from Greece and Turkey. This is in line with current observations concerning the presence of surface plastics in the Mediterranean basin. The Mediterranean Sea, being a semi-closed and shallow sea with high population density, is identified as one of the regions with the greatest accumulation of floating marine litter [61,62]. Plastic contamination has been documented in all the marine coastal areas considered in this study [47,63]. In particular, the Cilician Sea is one of the most contaminated coastal areas; in addition, the Gulf of Izmir in Turkey and the Bay of Thessaloniki in Greece can also be considered hot spots for the release of plastics into the marine environment [64]. The huge contamination in these areas is mainly attributed to an anthropogenic input linked to the high use of plastic by the local population, coupled with incorrect disposal and limited recycling (around 20% in Greece and 40% in Turkey) [65]. The different extent of plastic pollution observed in fish from the different origins might be related to the different magnitude of local contamination, even though other factors might significantly contribute to the plastic loads.

Plastics ingested by wild or farmed fish are closely related to their presence, distribution, and fate in the environment [66]. In aquaculture systems, in which location, structures, diet, etc. can be controlled, knowing the origin of the contamination can be very important to promote the environmental safety of the supply chain. Different sources of plastic pollution can be identified in aquaculture either from the external environment or from aquaculture processes. Plastics reach the marine environment through land-based sources and riverine systems collecting improper waste disposal, domestic and industrial sewage treatment plants, and agricultural productions [61]. Albeit with a lower input, the aquaculture environment can be contaminated also by plastics that originated from atmospheric deposition (nanoplastics and MPs) as well as from pelagic pollution [67]. Moreover, aquaculture practices generate a huge amount of plastic litter derived from several pieces of equipment made of plastics commonly used such as fishing gears, ropes, nets, buoys, and pipes [68]. In addition, processing (handling, transport, etc.) and packaging of fish imply the use of plastic trays, boxes, and disposables for workers that can also contribute to contamination [68]. A *non*-negligible amount of plastics is also introduced through contaminated feed, as have already been mentioned [69].

The qualitative characterization of plastics taken up by fish showed a similar typology of plastic polymers in fish from all the three countries. A prevalence of PEST and PA fibers was observed. Both polymers are secondary plastics of textile origin, so likely associated with land-based origin such as from the production of clothing and with the release from washing machines [70,71,72]. In addition, both kinds of polymers can also be released from different fishing and aquaculture tools, such as ropes and nets [73]. Also, PP is adopted in aquaculture for ropes, nets, tubes, and trays [73]. However, given the wide range of applications of PP, for instance in packaging, medical devices, and automotive, a land-based origin is also plausible for this plastic. Unlike other polymers, EP is not used in aquaculture, but EP particles might have a pelagic origin being released from ship painting [74].

As a final remark, a large portion of particles detected in fish was represented by man-made cellulose-based fibers and regenerated fibers (e.g., azlon). Indeed, these particles recently emerged as the most common anthropogenic debris found in aquatic ecosystems also able to induce adverse effects in aquatic biota [75,76,77,78,79,80], highlighting the importance of including this kind of particles in future monitoring studies.

## 5. Conclusions

The results of this study confirm the bioavailability of plastics to cultured marine fish, highlighting the need to thoroughly monitor this kind of contamination. Our data contribute to increasing the awareness of the fact that marine aquaculture systems are susceptible to plastic pollution introduced through multiple sources, and effective solutions to this problem are needed to promote the highest quality of cultured food, together with the adoption of best practices and measures aimed to reduce the release of plastic litter from aquaculture systems. It is also mandatory to sustain land-based and coastal management actions aimed to minimize the input of plastics into the marine environment.

## Figures and Tables

**Table 1:** State of the art regarding detection of plastics in seabass and seabream.

Matrix	Geographic Region	Extraction Technique	Instrumental Analysis	Limits of the Method	Range Concentration	Ref.
GIT of *D. labrax* and *S. aurata* (aquaculture)	Canary Islands Spain	Digestion in 10% KOH (*w/v*) at 60 °C for 24 h, filtration (50 µm mesh stainless-steel filters)	Visual sorting with microscope. MP characterization with FTIR	50 µm	Items/ind. Range: 0–23 Items length range: 69 µm–12.4 mm	[23]
Muscle of wild and farmed *S. aurata*	Tunisia	Mineralization with 65% nitric acid, extraction with dichloromethane (DCM), and dispersion in aluminum stubs for scanning electron microscope: “SEM Specimen Stubs”	Scanning electron microscopy coupled to a X energy dispersion detector (SEM-EDX)	<10 µm	Smallest and biggest median (IQR) diameter of MPs (1.8 and 2.5 μm). In *S. aurata* farmed mean MPs ± SD (p/g) 9.50 × 10^4^ ± 6.64 × 10^4^. Min-Max (p/g) 4.97 × 10^4^–21.20 × 10^4^. Median (IQR) 7.38 × 10^4^ (5.53–14.54 × 10^4^). Mean diameter ± SD (μm) 2.04 ± 0.30. Min-Max (μm) 1.7–2.4. Median (IQR) 1.9 (1.8–2.35)	[24]
GIT of *D. labrax* (aquaculture)	Canary Islands Spain	Digestion with 10% KOH (2 weeks, room temperature), filtration (25 μm mesh stainless-steel filters), stock in 10% EDTA (1 day)	Visual sorting with microscope. Larger particles (>200 μm) analyzed with FT-IR. Smaller particles (<200 μm) analyzed with a μ-FTIR	10 µm	0.6 ± 0.8–2.7 ± 1.85 particles/ind	[25]
GIT of *D. labrax* (aquaculture)	Mondego estuary Portugal	Digestion with 10% KOH (5 days, 60 °C), filtration (1.2 μm filter papers) desiccation at 60 °C	Visual sorting with stereomicroscope. MPs characterization with μ-FTIR	≤1 mm	38% of the fish ingested MP average of 1.67 ± 0.27 (SD) particles/ind. Average 3.41 ± 2.91 (SD) microplastics/ind of the individuals that had ingested MPs	[26]
GIT of *S. aurata* and *D. labrax* (aquaculture)	Turkey	Digestion with 20 mL of 30% H_2_O_2_ per gram under heating, addition of 400 mL NaCl solution (1.2 g/mL NaCl), and filtration (50 μm pore size filters)	Visual sorting with microscope. MP characterization with FTIR	50 µm (mesh size)	50% of seabream and 52% of seabass contaminated. Mean MP abundance in the GIT 1 ± 1.6 particles/ind. MP abundance in seabream 0.8 ± 1.1 particles/ind and 0.95 ± 1.1 particles/ind in seabass. Mean length of MPs 1.4 ± 1.3 mm	[27]
Flesh, gills and GIT of *D. labrax* (aquaculture)	Greece	Digestion with 30% H_2_O_2_ (24 h, 65 °C at 80 rpm, followed by 24–48 h at room temperature), filtration (5 μm pore size, 47 mm diameter cellulose membrane filter)	Visual sorting with stereomicroscope MP characterization with µ-FT-IR.	5 μm (pore size)	Incidence of contaminated fish 17%	[28]
GIT of *S. aurata* (aquaculture)	Italy and Croatia	Digestion with 10% KOH (48 h, 50 °C with oscillation), addition of hypersaline NaCl solution (15%), and filtration (glass fiber membrane with 1.5 mm and 0.7 mm pore size and 47 mm diameter).	Visual sorting with stereomicroscope. MP characterization with μ-FT-IR	240 μm (smallest detected)	0.21 (Fry) and 1.3 (adult) items/ind., respectively. 0.48 items/ind (Fry and adults). Fibers, ranging in size from 0.24 to 8.86 mm.	[29]
Flesh, gills, and GIT *D. labrax* (wild)	Portugal	Digestion with 10% KOH (24 h, 60 °C for flesh and GIT, 72 h, 40 °C for gills), filtration (glass-microfiber filter with 1.2 µm pore size).	Visual sorting with stereomicroscope. MP characterization with μ-FT-IR	<100 μm	42% of fish contaminated; 1.3 ± 2.5 MP items/ind. in the GIT 0.8 ± 1.4 MP items/ind. in gills and 0.4 ± 0.7 MP items/g in the dorsal muscle	[30]
GIT *S. aurata* (wild)	Egypt	Digestion with 10% potassium hydroxide (KOH), incubation 40 °C; filtration on 20 μm and nitrocellulose filter.	Visual sorting with stereomicroscope. MPs characterization with differential scanning calorimetry (DSC) and thermal gravimetric analysis (TGA).	>20 μm	93.3% of fish contaminated 38.3 ± 28.4 items/ind	[31]

IQR: interquartile range.

**Table 2:** Shape, size, dimension, color, and chemical composition (PP = polypropylene, PEST = polyester, EP = epoxy resin, PA = polyamide) of plastic particles found in fish.

Origin	Species	Organ	Shape	Dimension (mm)	Color	Chemical Composition
Turkey	*S. aurata*	GIT	Fragment	1.30	Green	PP
	*D. labrax*	GIT	Fiber	0.58	Black	PEST
	*D. labrax*	GIT	Fiber	0.28	Blue	EP
	*S. aurata*	GIT	Fiber	0.23	Black	PA
	*D. labrax*	GIT	Fiber	2.30	Black	PA
	*S. aurata*	muscle	Fiber	1.01	Black	PEST
	*D. labrax*	GIT	Fragment	0.27	Brown	PA
	*D. labrax*	GIT	Fragment	0.12	Black	PP
Greece	*S. aurata*	GIT	Fiber	1.62	Transparent	PEST
	*S. aurata*	GIT	Fiber	0.85	Transparent	PA
	*S. aurata*	GIT	Fiber	1.88	Transparent	PA
	*S. aurata*	liver	Fiber	1.30	Blue	PA
	*S. aurata*	GIT	Fiber	2.08	Black	PEST
	*S. aurata*	GIT	Fiber	1.09	Red	PA
	*S. aurata*	GIT	Fiber	1.59	Black	PEST
	*D. labrax*	muscle	Fragment	0.53	Blue	EP
	*D. labrax*	GIT	Fiber	0.50	Black	PEST
Italy	*D. labrax*	GIT	Fiber	3.34	Transparent	PEST

**Table 3:** State of the art regarding detection of plastics in fish from the Mediterranean Sea, with similar feeding habits of seabass and seabream.

Matrix	Geographic Region	Extraction Technique	Instrumental Analysis	Limits of the Method	Range Concentration	Ref.
GIT of *Boops boops*	Spain, France, Italy, and Greece	Digestion with hydrogen peroxide (H_2_O_2_ 15%), filtration under vacuum on fiber glass filters (pore size 1.2 μm)	Visual sorting with stereomicroscope. MP characterization with FTIR	1.2 μm	46.8% of positiveness, 1.17 ± 0.07 items/ind. 1–14 items per fish	[46]
GIT of 28 different species	Turkey	Digestion with 35% H_2_O_2_. Filtration with 26 μm zooplankton mesh	Visual sorting with stereomicroscope. MP characterization with FTIR	26 μm	58% of positiveness with average 2.36 items/ind.	[47]
GIT of 4 different demersal fish	Adriatic Sea	Digestion with 10% KOH, 48 h at 50 °C; separation with NaCl hypersaline solution; filtration under vacuum on GF/F fiber glass filters (0.7 μm). Staining with Nile red (9-diethylamino-5H-benzo[α]phenoxazine-5-one)	Visual sorting with stereomicroscope. MP characterization with μ-Raman spectroscopy	0.7 μm	57.5% of positiveness and up to 2.47 ± 2.99 items/ind.	[48]
GIT and gills of five demersal fish species	Southern Tyrrhenian Sea	No extraction. The GIT and the gills were inspected with the aid of a dissecting stereomicroscope	Visual sorting with stereomicroscope. MP characterization with ATR-FTIR and μ-Raman spectroscopy.	1 μm (Raman spatial resolution)	16.8% of positiveness with average 0.24 items/ind.	[49]
GIT of *Mullus barbatus* and *Merluccius merluccius*	North Tyrrhenian Sea, Adriatic Sea, and Ionian Sea	Extraction with 10% of KOH solution 1/3 *v.v.* incubated at 60 °C for 6 h after 15 min sonication, filtration on glass fiber filters (1.6 μm mesh)	Visual sorting with stereomicroscope. Test with *hot needle* technique. MP characterization with FTIR	>100 μm	23.3% of positiveness, range 8.3–48% Average 1.38 plastics/ind.	[8]
GIT of *Scyliorinus canicula* and *Mullus barbatus*	Alboran Sea	Digestion with 10% KOH, homogenization, filtration on 150 μm sieve	Visual sorting with stereomicroscope (ultraviolet light or through a scanning electron microscope). MP characterization with μFTIR	150 μm	9.8 and 32.7% of positiveness (7 and 24 fibers/ind)	[50]
GIT of two congener species of seabreams: *Pagellus erythrinus* and *P. bogarave*	Tyrrhenian Sea	Manual extraction	Visual sorting with stereomicroscope. MP characterization with FTIR and Raman spectroscopy	Not reported	12.5% of positiveness	[51]
GIT of *Mullus barbatus* and *Umbrina cirrosa*	Central Tyrrhenian Sea	Digestion with 5% HNO_3_ + 15% H_2_O_2_, incubation 40 °C; filtration on 2.7 μm glass microfiber membrane	Visual sorting with stereomicroscope. MP characterization with FTIR	2.7 μm	90% of positiveness, 3.4 ± 1.9 items/ind. and 1–8 as range	[52]
GIT of commercial fish	Egypt	Digestion with 10% potassium hydroxide (KOH), incubation 40 °C; filtration on 20 μm and nitrocellulose filter	Visual sorting with stereomicroscope. MP characterization with differential scanning calorimetry (DSC) and thermal gravimetric analysis (TGA)	>20 μm	91.8 ± 8.4% of positiveness and an average of 11.7 ± 9.5 items/ind.	[31]
GIT and gills of demersal fish	Turkey	Digestion with 30% H_2_O_2_, filtration on 50 μm pore size filter	Visual sorting with microscope. MP characterization with FTIR	>50 μm	85% of positiveness	[53]
Different species	Egypt	Digestion with 10% H_2_O_2_, incubation 50 °C; second digestion with 30% H_2_O_2_, filtration on 1 mm and 300 μm sieve	Visual sorting with microscope. MP characterization with ATR-FTIR.	>300 μm	58% of positiveness, 2.36 items/ind.	[54]

## Data Availability

Data are available upon request.

## Supplementary Materials

The following supporting information can be downloaded at: https://www.mdpi.com/article/10.3390/jox13040040/s1, Table S1: List of samples and tissue analyzed; Table S2: Anthropogenic particles found in blanks; Table S3: Anthropogenic particles found in fish.
