# Supplementary material for: Plastic Contamination in Seabass and Seabream from Off-Shore Aquaculture Facilities from the Mediterranean Sea
Source: J Xenobiot. 2023 Oct 25;13(4):625–40. doi: 10.3390/jox13040040 (PMC10660701; doi:10.3390/jox13040040)
Supplement: Supplementary file 1 [file jox-13-00040-s001.zip › Table S1.pdf]

Species	Analyzed matrices	Origin	plastics
Seabream	GIT	Greece	1
	muscle		
Seabass	GIT	Greece	
Seabass	GIT	Italy	
Seabream	GIT	Italy	
	Liver		
	muscle		
Seabream	GIT	Greece	
	muscle		
Seabass	GIT	Greece	
Seabass	GIT	Greece	
	muscle		
Seabass	GIT	Turkey	
Seabream	GIT	Greece	
Seabass	GIT	Greece	
Seabream	GIT	Greece	2
	Liver		
	muscle		
Seabass	GIT	Italy	
Seabream	GIT	Turkey	1
	Liver		
	muscle		
Seabream	GIT	Greece	
	muscle		
Seabass	GIT	Turkey	2
	muscle		
	Liver		
Seabream	GIT	Greece	
	Liver		1
Seabass	GIT	Greece	
	Liver		
Seabream	GIT	Turkey	
Seabass	GIT	Italy	1
Seabream	GIT	Turkey	1
	muscle		
	Liver		
Seabream	GIT	Greece	
Seabass	GIT	Greece	
Seabass	GIT	Greece	
Seabream	GIT	Italy	
Seabass	GIT	Turkey	
Seabream	GIT	Greece	3
Seabass	GIT	Turkey	1
Seabass	GIT	Greece	
	muscle		1
Seabream	GIT	Turkey	
	muscle		1
Seabass	GIT	Greece	1
Seabass	GIT	Turkey	2
Seabream	GIT	Greece	
	muscle		
Seabream	GIT	Greece	
	muscle		
Seabream	GIT	Turkey	

Table S1: List of samples and tissue analyzed.
