# Supplementary material for: Plastic Contamination in Seabass and Seabream from Off-Shore Aquaculture Facilities from the Mediterranean Sea
Source: J Xenobiot. 2023 Oct 25;13(4):625–40. doi: 10.3390/jox13040040 (PMC10660701; doi:10.3390/jox13040040)
Supplement: Supplementary file 1 [file jox-13-00040-s001.zip › Table S2.pdf]

Sample	Shape	Size (mm)	Colour	Chemical composition	Score
B13	fiber	0.76	transparent	cellulose	0.73
B29	fiber	0.68	red	cellulose	0.86
B33	fiber	1.25	transparent	cellulose	0.76
B14	fiber	1.29	violet	cellulose	0.80
B18	fiber	1.19	blue	cellulose	0.73
B39	fiber	1.13	blue	celloluse	0.79
B31	fiber	2.41	black	cellulose	0.81
B15	fiber	2.56	brown	rayon	0.80

Table S2: Anthropogenic particles found in blanks.
