# Supplementary material for: Plastic Contamination in Seabass and Seabream from Off-Shore Aquaculture Facilities from the Mediterranean Sea
Source: J Xenobiot. 2023 Oct 25;13(4):625–40. doi: 10.3390/jox13040040 (PMC10660701; doi:10.3390/jox13040040)
Supplement: Supplementary file 1 [file jox-13-00040-s001.zip › Table S3.pdf]

Table S3: Anthropogenic particles found in fish.

Fish	Origin	Organ	Shape	Size (mm)	Colour	Chemical composition	Score
S. aurata	Greece	GIT	fiber	1.62	transparent	polyester	0.75
			fragment	0.52	red	fat	0.85
			fragment	0.26	brown	fat	0.71
		muscle	fiber	0.96	red	cellulose	0.89
			fiber	0.65	blue	cellulose	0.93
D. labrax	Greece	GIT	fragment	0.42	black	fat	0.74
D. labrax	Italy	GIT	fiber	0.34	black	cellulose	0.70
			fiber	0.58	transparent	cellulose	0.77
			fiber	0.28	black	cellulose	0.79
			fiber	2.43	black	cellulose	0.80
			fiber	0.64	grey	cellulose	0.79
S. aurata	Italy	GIT	fiber	0.87	blue	cellulose	0.75
			fiber	0.41	blue	aluminum sulfate	0.82
			fiber	0.37	blue	natural polyamide	0.84
		liver	fiber	2.27	transparent	cellulose	0.82
			fiber	0.54	red	cellulose	0.72
D. labrax	Greece	GIT	fiber	0.97	black	fat	0.7
			fiber	0.50	transparent	fat	0.73
			fiber	0.61	black	cellulose	0.72
D. labrax	Turkey	GIT	fragment	0.11	black	fat	0.72
			fiber	0.24	blue	natural polyamide	0.75
			fiber	0.33	violet	cellulose	0.70
			fiber	0.25	transparent	cellulose	0.70
S. aurata	Greece	GIT	fiber	0.40	transparent	cellulose	0.70
D. labrax	Greece	GIT	fiber	0.83	black	natural polyamide	0.83
			fiber	2.26	red	cellulose	0.83
			fiber	0.72	black	cellulose	0.74
			fiber	1.51	red	cellulose	0.73
			fiber	1.54	black	cellulose	0.77
S. aurata	Greece	GIT	fiber	0.85	transparent	polyamide	0.93
			fiber	1.88	transparent	polyamide	0.87
D. labrax	Italy	GIT	fiber	1.01	blue	cellulose	0.75
			fiber	0.66	black	natural polyamide	0.74
S. aurata	Turkey	GIT	fragment	1.30	green	polypropylene	0.97
		muscle	fiber	1.92	black	cellulose	0.76
S. aurata	Greece	GIT	fiber	0.76	transparent	cellulose	0.76
		muscle	fiber	1.50	transparent	cellulose	0.85
			fragment	0.05	red	fat	0.81
D. labrax	Turkey	GIT	fiber	0.58	black	polyester	0.80
			fiber	0.28	blue	epoxy resin	0.71
			fragment	0.22	yellow	natural polyamide	0.77
		liver	fiber	0.39	brown	cellulose	0.89
			fiber	1.07	transparent	cellulose	0.78
			fiber	0.39	blue	cellulose	0.74
			fiber	0.19	blue	cellulose	0.74
			fiber	0.62	violet	cellulose	0.73
S. aurata	Greece	liver	fiber	1.30	blue	polyamide	0.69
			fiber	0.82	brown	cellulose	0.79
			fiber	0.63	black	cellulose	0.77
D. labrax	Greece	liver	fiber	2.59	transparent	cellulose	0.75
			fiber	0.32	transparent	natural polyamide	0.78
			fiber	2.20	blue	natural polyamide	0.76
			fiber	1.31	transparent	cellulose	0.88
			fiber	1.07	transparent	cellulose	0.85
			fiber	1.46	transparent	cellulose	0.82
		GIT	fiber	0.68	blue	cellulose	0.84
			fiber	0.68	violet	cellulose	0.78
			fragment	0.25	transparent	cellulose	0.79
			fiber	0.63	blue	cellulose	0.76
			fiber	1.82	blue	cellulose	0.73
			fiber	0.73	blue	cellulose	0.82
			fiber	0.63	red	cellulose	0.70
S. aurata	Turkey	GIT	fiber	4.07	transparent	cellulose	0.70
			fiber	1.72	black	cellulose	0.80
D. labrax	Italy	GIT	fiber	2.65	red	cellulose	0.72
			fiber	3.34	transparent	polyester	0.93
S. aurata	Turkey	GIT	fiber	0.37	black	cellulose	0.79
			fiber	0.31	violet	cellulose	0.81
			fiber	0.23	black	polyamide	0.73
			fiber	0.20	black	natural wax	0.71
			fiber	0.78	black	natural wax	0.75
		liver	fiber	0.52	brown	natural wax	0.74
D. labrax	Greece	GIT	fiber	1.81	transparent	cellulose	0.85
			fiber	1.46	black	cellulose	0.83
			fiber	1.04	red	cellulose	0.84
S. aurata	Greece	GIT	fiber	2.08	black	polyester	0.91
			fiber	0.54	black	cellulose	0.72
			fiber	1.09	red	polyamide	0.71
			fiber	0.39	brown	natural polyamide	0.79
			fiber	0.86	black	cellulose	0.70
			fiber	1.59	black	polyester	0.81
D. labrax	Turkey	GIT	fiber	0.41	red	natural wax	0.73
			fiber	0.18	black	natural polyamide	0.78
			fiber	0.47	red	cellulose	0.71
			fiber	2.30	black	polyamide	0.74
D. labrax	Greece	GIT	fiber	1.18	orange	natural polyamide	0.76
			fragment	0.13	brown	natural polyamide	0.70
		muscle	fragment	0.53	blue	epoxy resin	0.92
S. aurata	Turkey	GIT	fiber	0.30	black	cellulose	0.71
		muscle	fiber	1.01	black	polyester	0.91
			fragment	1.23	orange	natural wax	0.85
			fragment	1.01	red	natural polyamide	0.72
D. labrax	Greece	GIT	fiber	0.53	black	cellulose	0.73
			fiber	0.50	black	polyester	0.8
			fiber	0.20	blue	cellulose	0.88
			fiber	1.03	black	cellulose	0.83
			fiber	0.37	transparent	cellulose	0.72
S. aurata	Greece	GIT	fiber	0.18	blue	cellulose	0.80
			fiber	0.65	blue	cellulose	0.89
D. labrax	Turkey	GIT	fiber	0.76	transparent	cellulose	0.88
			fragment	0.27	brown	polyamide	0.74
			fiber	2.61	black	cellulose	0.86
			fiber	0.57	blue	cellulose	0.79
			fiber	1.85	transparent	cellulose	0.80
			fragment	0.12	black	polypropylene	0.87
			fiber	3.42	black	cellulose	0.90
			fragment	0.22	orange	magnesium silicate	0.80
			fiber	1.05	blue	cellulose	0.77
S. aurata	Turkey	GIT	fiber	0.30	transparent	cellulose	0.76
			fiber	0.64	brown	cellulose	0.72
S. aurata	Greece	GIT	fiber	1.04	black	cellulose	0.73
